# Coping with COVID in corrections: a qualitative study among the recently incarcerated on infection control and the acceptability of wastewater-based surveillance

**DOI:** 10.1186/s40352-023-00205-0

**Published:** 2023-02-07

**Authors:** Lindsey R. Riback, Peter Dickson, Keyanna Ralph, Lindsay B. Saber, Rachel Devine, Lindsay A. Pett, Alyssa J. Clausen, Jacob A. Pluznik, Chava J. Bowden, Jennifer C. Sarrett, Alysse G. Wurcel, Victoria L. Phillips, Anne C. Spaulding, Matthew J. Akiyama

**Affiliations:** 1 Albert Einstein College of Medicine, Montefiore Medical Center, Bronx, NY USA; 2 Rollins School of Public Health, Emory University, Atlanta, GA USA; 3 Center for the Study of Human Health, Emory University, Atlanta, GA USA; 4 Tufts Medical Center, MA Boston, USA

**Keywords:** Prison, Jail, COVID-19, Pandemic, SARS-CoV-2, Incarcerated, Justice-involved individuals, Wastewater, Surveillance

## Abstract

**Background:**

Correctional settings are hotspots for SARS-CoV-2 transmission. Social and biological risk factors contribute to higher rates of COVID-19 morbidity and mortality among justice-involved individuals. Rapidly identifying new cases in congregate settings is essential to promote proper isolation and quarantine. We sought perspectives of individuals incarcerated during COVID-19 on how to improve carceral infection control and their perspectives on acceptability of wastewater-based surveillance (WBS) accompanying individual testing.

**Methods:**

We conducted semi-structured interviews with 20 adults who self-reported being incarcerated throughout the United States between March 2020 and May 2021. We asked participants about facility enforcement of the Centers for Disease Control and Prevention (CDC) COVID-19 guidelines, and acceptability of integrating WBS into SARS-CoV-2 monitoring strategies at their most recent facility. We used descriptive statistics to characterize the study sample and report on acceptability of WBS. We analyzed qualitative data thematically using an iterative process.

**Results:**

Participants were predominantly Black or multiple races (50%) and men (75%); 46 years old on average. Most received a mask during their most recent incarceration (90%), although only 40% received counseling on proper mask wearing. A quarter of participants were tested for SARS-CoV-2 at intake. Most (70%) believed they were exposed to the virus while incarcerated. Reoccurring themes included (1) Correctional facility environment leading to a sense of insecurity, (2) Perceptions that punitive conditions in correctional settings were exacerbated by the pandemic; (3) Importance of peers as a source of information about mitigation measures; (4) Perceptions that the safety of correctional environments differed from that of the community during the pandemic; and (5) WBS as a logical strategy, with most (68%) believing WBS would work in the last correctional facility they were in, and 79% preferred monitoring SARS-CoV-2 levels through WBS rather than relying on just individual testing.

**Conclusion:**

Participants supported routine WBS to monitor for SARS-CoV-2. Integrating WBS into existing surveillance strategies at correctional facilities may minimize the impact of future COVID-19 outbreaks while conserving already constrained resources. To enhance the perception and reality that correctional systems are maximizing mitigation, future measures might include focusing on closer adherence to CDC recommendations and clarity about disease pathogenesis with residents.

## Background

The United States (U.S.) reports the highest incarceration rate in the world (ICPR 2022). On any given day, 2.2 million individuals are detained or imprisoned (Sawyer 2021), whether in prisons which house individuals who are sentenced to greater than 1 year or jails housing individuals detained pre-trial or with sentences shorter than one year (Spaulding et al. 2011). Predictably, prisons and jails emerged as epicenters of COVID-19 outbreaks early in the pandemic (Akiyama et al. 2020; Franco-Paredes et al. 2020; Gandhi et al. 2020; Reinhart and Chen 2020)with evidence of sustained transmission in the spring and summer of 2021 (Akiyama et al. 2020; Jensen 2021; Park 2021; Epting et al. 2021). Moreover, co-morbid conditions place many individuals involved in the legal system at an increased risk of severe illness and mortality from SARS-CoV-2 (Hawks et al. 2020), therefore they have continued to reap the negative effects of infection at much higher rates than the surrounding community.

The high turnover of the U.S. correctional system(Spaulding et al. 2011; Zeng 2021)coupled with the congregate nature of carceral settings, instances of substandard healthcare (Akiyama et al. 2020; CDC 2021; Kinner et al. 2020)and limited access to sufficient cleaning supplies and personal protective equipment (PPE)(Shortell 2020; Solis et al. 2020), particularly early in the pandemic (Akiyama et al. 2020; Kinner et al. 2020; Franco-Paredes 2021), have contributed to the above average infection rates in an already marginalized population. The physical, social and biological factors that put justice-involved individuals at an increased risk for contracting COVID-19 and experiencing higher rates of morbidity and mortality (Chan et al. 2021; Nowotny et al. 2020; Pettus-Davis 2021) suggest that new strategies are needed to rapidly identify outbreaks that will avoid placing undue burden on correctional staff and residents.

Multifaceted strategies to mitigate the spread of SARS-CoV-2 were unveiled in the spring of 2020 (Hawks et al. 2020; Kinner et al. 2020; Wurcel et al. 2020). Early approaches included reducing population density, which has been demonstrated to have population-level public health benefits (Reinhart and Chen 2021; Vest et al. 2021), and minimizing movement of the individuals who remained (Hawks et al. 2020; Malloy 2020; Zawitz 2020; Collica-Cox 2020; Rao 2020; Jiménez et al. 2020; Macmadu 2021; Simpson 2020; Tompkins et al. 2021). Many facilities have integrated mass screening of asymptomatic residents and employees (Hagan et al. 2020). As of late, most facilities now offer vaccines to these two groups. However vaccine hesitancy (Langer 2020; Iverac 2021; Stern et al. 2021)coupled with the emergence of more concerning SARS-CoV-2 variants, demonstrate the need for not only increased vigilance but also improved surveillance tools in correctional settings (Spaulding et al. 2011).

Early on, investigators advocated for correctional institutions to implement wastewater-based surveillance (WBS) to monitor for the presence of SARS-CoV-2 in the wastewater (Nghiem 2020; Wang et al. 2020). The approach of WBS has been used in the past to monitor for disease on the community-level such as detecting*Salmonella* Typhi, *Vibrio cholerae*, and poliovirus (Matrajt et al. 2020; Sears et al. 1984; Barrett et al. 1980; Tao et al. 2010). Early in the COVID-19 pandemic, this approach was adopted for monitoring in various municipalities in Connecticut and Spain (Peccia 2020; Randazzo et al. 2020)and then on an institutional level such as University of Arizona and University of California San Diego (Kreier 2021; Betancourt et al. 2021). The literature suggests that while WBS is often accepted by the general community for routine surveillance unrelated to SARS-CoV-2, there are ethical concerns when WBS is conducted on a smaller scale such as within correctional facilities or workplaces (Scassa 2022; Hall et al. 2012; LaJoie et al. 2022). For example, if WBS identifies that illicit substances are being used in a correctional facility, there is a concern these findings could prompt policy changes that could adversely affect residents including limiting or eliminating visiting hours in efforts to curb drug smuggling (Hall et al. 2012). While WBS may be a critical tool for correctional facilities, little was known about the acceptability of this surveillance strategy among justice-involved population. Taking a participatory approach by surveying justice-involved individuals with lived experience of incarceration during COVID-19 pandemic not only allows us to fill in existing gaps in knowledge about implementing WBS in correctional systems, but also serves to empower residents and individuals with lived experience to initiate change (Farrell 2021). The objective of this study was to gain understanding of how to improve carceral infection control from individuals with lived experience of incarceration during COVID-19 pandemic. In addition, this study attempted to collect knowledge and attitudes towards various SARS-CoV-2 surveillance strategies including WBS.

## Methods

### Setting and participants

For this qualitative study, we conducted a survey followed by open-ended interviews with 20 formerly incarcerated individuals in Georgia, Massachusetts, Connecticut, and New York from June 2021 through September 2021. Our survey was designed to assess disease monitoring and infection control in jails and prisons from the perspective of individuals who were incarcerated during the COVID-19 pandemic, and to solicit ideas how to improve correctional infection control. Participants were considered eligible if they: (1) were at least 18 years old; (2) English-speaking, and (3) self-reported being incarcerated in a US carceral setting at least once between March 2020 and June 2021, prior to the onset of the Delta and Omicron surges.

Participants were referred to the study by partner organizations that work directly with formerly incarcerated individuals. Individuals were also actively recruited by research staff onsite at corrections-focused community-based organizations.

### Data collection

We developed a quantitative questionnaire based on a literature review to elicit perceptions of being incarcerated during the COVID-19 pandemic. We assessed demographic characteristics, recent history of incarceration, history of treatment for substance use disorder, infection control and surveillance strategies and the impact of COVID-19 during incarceration (access to COVID-19 testing; social distancing precautions; medical care) using structured questions and collected via Qualtrics (Seattle, WA). Questions and responses are listed in Tables 1, 2 and 3. For the qualitative portion, participants were asked open-ended questions to ascertain facility COVID-19 guidelines, such as whether they believe COVID-19 is a public health problem for individuals in their community, the perceived threat of COVID-19 exposure while incarcerated, as well as questions related to the acceptability of using WBS to monitor for COVID-19 infection in correctional facilities.

**Table 1:** Sociodemographic characteristics (*n* = 20)

Characteristics	*N* (%) or median [IQR or range]
Age (median, IQR)	45.5 [35.75–52.25]
Male	15 (75.0%)
Race	
* White*	7 (35.0%)
* Black or African American*	8 (40.0%)
* Other*	3 (15.0%)
* Multiple Races*	2 (10.0%)
Hispanic	4 (20.0%)
Education	
* Did not finish high school*	1 (5.0%)
* Graduated from high school*	5 (25.0%)
* GED*	4 (20.0%)
* Finished some college*	7 (35.0%)
* Graduated from college*	3 (15.0%)
Clinical characteristics	
* Have gone to AA/NA/etc. for drug or alcohol use in the last 5 years*	10 (50.0%)
* Ever diagnosed with a mental problem*	15 (75.0%)
* Enrolled in chronic care clinic at last correctional facility*	3 (15.0%)
Justice involvement during pandemic	
* Median number of months incarcerated between March 2020-May 2021 [range]*	7 [1–15]
* Median number of visits since March 2020 [range]*	1 [1–2]
* Median number of facilities since March 2020 [range]*	1.6 [1–4]
* Drug or alcohol-related charges*	5 (25%)
Type of correctional facility at last stay	
* Prison*	10 (50.0%)
* Jail*	10 (50.0%)
State of last correctional facility	
* Connecticut*	3 (15.0%)
* Georgia*	10 (50.0%)
* Massachusetts*	2 (10.0%)
* New York*	5 (25.0%)

**Table 2:** Self-reported COVID-19 precautions during last incarceration and vaccine acceptability (*n* = 20)

COVID-19-related precautions	N (%)
Provided a mask	18 (90.0%)
Educated on proper mask-wearing	8 (40.0%)
* Verbally*	7 (87.5%)
* Video*	1 (12.5%)
Educated on social distancing	7 (35.0%)
* Verbally*	6 (75.0%)
* Video*	0 (0.0%)
* Both*	1 (12.5%)
Educated on proper hand hygiene	7 (35.0%)
* Verbally*	7 (87.5%)
* Video*	0 (0.0%)
* Both*	0 (0.0%)
Signs about precautions	10 (50.0%)
Tested at intake with nasal swab	5 (25.0%)
* Received results (n = 5)*	5 (100.0%)
* Tested positive (n = 5)*	1 (20.0%)
* Isolated with other COVID-19 cases (n = 5)*	1 (20.0%)
To the best of my knowledge, I was exposed to COVID-19	14 (70.0%)
I was informed if individuals in my unit tested positive	8 (40.0%)
I was placed in quarantine at entry	8 (40.0%)
I was placed in quarantine after an exposure	8 (40.0%)
COVID-19 vaccination status	
* I was vaccinated at my most recent facility*	7 (35%)
* I was vaccinated in the community (post-release)*	7 (35%)
* No, I have not been vaccinated*	6 (30%)
How likely are you to accept the vaccine (*n* = 6)?	
* Extremely likely*	2 (33.3%)
*Somewhat likely*	1 (16.7%)
* Somewhat unlikely*	1 (16.7%)
* Extremely unlikely*	2 (33.3%)
Which vaccine did you receive (*n* = 14)?	
* Pfizer*	4 (28.6%)
* Moderna*	5 (35.7%)
* Johnson and Johnson*	5 (35.7%)

**Table 3:** Water-based surveillance acceptability

	N (%)
Do you think this method of monitoring for COVID-19 would work in the last jail/prison you were in? (*n* = 19)	
* Yes*	13 (68.4%)
* No*	0 (0.0%)
* Maybe*	6 (31.6%)
Which would you prefer, wastewater testing or just individual testing? (*n* = 19)	
* Wastewater testing*	15 (78.9%)
* Just individual testing*	4 (21.1%)

Given COVID-19 social distancing precautions, most interviews were conducted via telephone or teleconferencing – three were conducted in person – and consent was obtained orally. Trained research staff members conducted a brief screening to determine participants’ eligibility for the study. If eligible, research staff reviewed the consent form and once consent was obtained, research staff administered the full survey. Participants first responded to the quantitative survey in which responses were entered in Qualtrics by research staff. Following completion of the quantitative survey, research staff conducted the qualitative interview in which responses were audio-recorded. All responses were stored under a code unique to the participants to ensure anonymity. Halfway through the interview participants watched a 3-minute video about using WBS to detect SARS-CoV-2 in a university setting. Altogether, the survey lasted between 35 and 40 min. Participants received a $30 electronic gift card upon completion of the interview. All study protocols were separately reviewed and approved by the [redacted and included in title page] Institutional Review Boards.

### Data analysis

We used descriptive statistics to characterize the study sample. Quantitative questions were posed about the acceptability of WBS. Frequencies and proportions were calculated for categorical data, and we calculated means, medians, interquartile ranges (IQR) or ranges, and standard deviations for continuous data.

Once all interviews were conducted and transcribed we analyzed qualitative data in an iterative process using a thematic analysis (Boyzatis 1998) to describe facility-wide COVID mitigation strategies and the related structural barriers to following guidelines, the influence of peers in regards to receiving information about mitigation strategies and acceptability of WBS to monitor for SARS-CoV-2. Four investigators (L.R., L.P, L.S. and P.D.) developed a coding scheme to categorize common themes that emerged upon reading of the first five transcripts. Discrepancies in initial coding were discussed and resolved by consensus to develop a final coding scheme; all transcripts were then independently coded by at least two coders using DeDoose software (Hermosa Beach, CA). The coding team subsequently discussed content by code, examining relationships between codes, and using the constant comparative method to identify, refine, and consolidate emergent themes. Given the logistics surrounding interviews including the time to transcribe and the geographic region from which we recruited, the team was unable to assess data saturation during the period of data collection, however during our coding meetings no further information emerged, allowing us to conclude that thematic saturation was achieved.

## Results

The 20 participants were predominately male (75%) with an average age of 46 years, ranging from 25 to 68 years old (Table 1). Most identified either as Black or African American (40%) or White (35%), and non-Hispanic (80%). Regarding their most recent period of incarceration, half of the participants were housed in a Georgia facility (50%) and a quarter in New York (25%), followed by Connecticut (15%) and Massachusetts (10%). Participants were incarcerated for 7 months during the pandemic on average, ranging from 1 to 15 months.

Regarding COVID-19 precautions, most participants reporting that while they received a mask during their most recent incarceration (90%), the majority were not educated on how to wear masks properly (60%) or on social distancing or proper hand hygiene (65%) (Table 2).

Only a quarter reported being tested for COVID-19 at intake with a nasal swab; however, some were already incarcerated prior to the onset of the pandemic in their region. Most participants (70%) believe they were exposed to COVID-19 while incarcerated, either because they were informed by prison staff there was a case in their unit or dorm or because individuals in their living space who were sick were removed from the unit. Despite this, only 40% of those individuals reported being quarantined after an exposure.

Most participants were vaccinated (70%); seven received the vaccine while incarcerated and seven were vaccinated after incarceration in the community. Among the six participants who were not vaccinated at the time of the interview, half reported they were somewhat or extremely likely to get the vaccine in the future.

Our coding tree included five themes: (1) Correctional facility environment leading to a sense of insecurity; (2) Perceptions that punitive conditions in correctional settings were exacerbated by the pandemic; (3) Importance of peers as a source of information about mitigation measures; (4) Discordant perceptions on the safety of correctional settings compared with the community during the pandemic; and (5) WBS as a logical strategy.

### Correctional facility environment leading to a sense of insecurity

All participants reflected on how their facility implemented the Centers for Disease Control and Prevention (CDC) guidelines during the pandemic to reduce the risk of residents spreading and contracting SARS-CoV-2. Often the adoption was incomplete. Officers may have had ample access to PPE while only one disposable mask could have been issued per jail entrant. Changes in day-to-day activities may have been put in place ostensibly to keep residents safe, but they felt just the opposite. For some, the sense of insecurity was due to the congregate nature of carceral settings, while for others it was the perceived failure of the facility to provide adequate resources.

#### Congregate nature of carceral settings as a barrier to following guidelines

Many participants noted that the layout of correctional facilities, such as living in close quarters with more than 100 people and eating together in the dining hall made it difficult to follow the COVID-19 guidelines that were implemented at their facility. One participant reflected on the difficulty of following social distancing guidelines throughout their day.

*“When you’re in a dormitory, you’re literally two feet away from the next person’s cube. So, how much is that social distancing. … I mean, you’re living in a dormitory with at least 50 people and … it’s just impossible to be far away from anyone, especially six feet. You’re literally three feet away from these people. So, it was hard, it was hard ... our safety was always jeopardized, because we were crowded.” –* Participant R, a 39-year-old male.

Other participants expressed similar concerns related to population density exacerbating the spread of the virus, especially when infected residents were sheltered in place rather than placed in an isolation unit.

*“Trying to quarantine was still inhumane due to the fact that if they knew people were sick, they quarantined the whole dorm and you know, that’s a 120-man dorm and so, if somebody is in there sick, you’re stuck in that same dorm, you can’t go anywhere. So, now it’s spreading. So, you pretty much have to be there and deal with it.” –* Participant C, a 31-year-old male.

For another participant, it wasn’t just the spatial constrictions that made it difficult to follow guidelines. Many participants reported their facility failed to provide proper education to residents on mask-wearing (60%), social distancing (65%), proper hand hygiene (65%). Moreover, these guidelines were not always enforced among staff:

*“They were not social distancing. And in their defense, it was kind of hard when you have three women or four women in one room, there’s no such thing as social distance. … Education would definitely help… no one knew why they had to wear the face mask or why they had the social distance or how important washing their hands was. … You want the inmates to stay six feet apart and wear the mask, but you have the staff coming through for count with no mask on coughing in the room.”* Participant B, a 36-year-old female.

For some the lack of adherence to guidelines was more evident among the medical staff, while for many others it was the correctional officers’ (CO) non-adherence that fostered feelings of insecurity.

*“So I do think the medical staff was a big part of it. Not wearing masks themselves, you know, when the offender sees that the nursing staff, you know, they make statements, and saying I don’t need to wear a mask I have full immunity, offenders are going to follow that. So, you have several nurses just didn’t adhere to the policy.”* - Participant M, a 46-year-old female.

Similar concerns related to the COs were expressed by another participant:

*“Well, I would wear a mask because in truth COs they caught it, and even when they came back, they weren’t wearing masks… They hang by a desk they are talking like they are at the restaurant is down at the corner. You know they are sitting there in groups. They were not social distancing. And they got it [COVID-19]. ….”* - Participant G, a 63-year-old male.

#### Limited resources confer a lack of safety

In addition to feeling unsafe because staff failed to follow guidelines themselves, for many participants inadequate access to resources such as cleaning supplies and mask provision conferred a sense of insecurity as well.

*“And they all just, you know, ‘Wear your mask,’ or just you know basically they told, well, they wouldn’t give us a mask but what they knew we were doing is we were taking t-shirts and just basically making our own masks. And so, that’s when they realized that we were more serious about it than they were...”* – Participant L, a 45-year-old female.

The feeling to make do with what was available was mirrored by other participants. One resident recalled making masks out of socks and described other extreme measures such as stealing bleach in order to have adequate disinfectant supplies:

*“Masks weren’t even distributed at that time. We had taken socks ourselves and sewn them to make a mask and cut holes in it for ears, you know. … We stole it [bleach]. The cleaning, I worked in laundry so I had access to like the pure bleach, and I would bring it in a bottle and put water in it because it was so concentrated that it would eat through the soft plastic. So, I would put water in it and bring it back to the room. We had a disposable rag and paper towels that my other roommate worked in cleaning the staff’s bathrooms, so she would take out their paper towels. That was stolen too. So, you just wanted to make sure you were clean and safe from anything.”* – Participant B, a 36-year-old female.

Given these experiences, many participants reflected on what could have been done better at the facility-level. In addition to increasing the provision of resources such as masks and cleaning supplies, suggestions included mandating testing upon entry, implementing more thorough screening procedures, educating residents on the virus and how it spreads, as well as informing individuals if they’ve been exposed to the virus.

### Perceptions that punitive conditions in correctional settings were exacerbated by the pandemic

Participants discussed how they often felt a lack of compassion from correctional staff members prior to the pandemic and that during the COVID-19 pandemic, these feelings were perpetuated by the actions taken at the facility-level and by correctional staff in response to infection control guidelines. For many, feelings were associated with a lack of respect and compassion, as well as being kept in the dark about the effect of the pandemic on the outside community.

In some instances where staff followed COVID-19 guidelines, residents perceived these actions as insensitive:

*“They wore masks. They just had us like animals in a cell, you know? They stood far back away from us.”* – Participant K, a 53-year-old male.

Similarly, others expressed feelings of helplessness perpetuated by a lack of adherence to safety precautions exhibited by facility staff.

*“Honestly, I don’t think they actually cared about the guidelines, because there were officers and I don’t want to sit there and like, bash people or, belittle anyone but in reality, they had a non-caring attitude. I mean, there were officers that made comments as if I’m sick and I got the COVID I hope I give it to everyone. Like they didn’t care, they didn’t care at all, some officers, not all, some. That was their attitude towards that whole situation.”* – Participant R, a 39-year-old male.

In addition to the feeling helpless from the COs’ actions, some also expressed frustration towards how their facility distributed PPE; equipping staff with the necessary PPE but failing to do the same for residents:

*“They have taken on this responsibility to oversee us and that that’s part of taking on responsibility of the job that you took instead of just dismissing it and making sure your staff is okay, like the staff had masks. And we knew it was masks that came from the facility because they all had [facility emblem] on it. So, we knew this was a facility, you can’t be passing out masks, but we didn’t understand why you did not you know do the same for us.”* – Participant L, a 45-year-old female.

Inequity in resource distribution was also evident at this participant’s facility where staff were provided hazmat suits while residents remained mask-less:

*“Before they let me out, they had to take my temperature and things like that. Even though that it was through a glass door because we didn’t have no masks. We didn’t have no masks. They did it through like a glass. They had masks on, they had hazmat suits basically.”* – Participant K, a 53-year-old male

#### Delay in receiving information

Besides not receiving resources, participants also reported not being informed about the pandemic from prison staff and having to rely on the television for information.

*“I mean, you know, the TV told us. The staff would come out and tell us nothing. We had to listen to the TV. … They didn’t have no group of experts or professionals to come out and educate us on methods. The only thing that was is the TV told you one thing and you had to adhere to what TV said.”* - Participant I, a 68-year-old male.

In addition to not being informed about mitigation measures, only 40% of participants reported being informed if those around them tested positive.

*“No, they didn’t say anything [if someone tested positive]. … They just packed them up like they were getting released. They didn’t tell anybody anything. And when I got yanked the next morning, I thought I was just going to court or something. So, they didn’t tell anybody in there. No. It’s all hush hush and they’re trying to, well, I’m just assuming they were just covering their asses.”* – Participant Q, a 56-year-old male.

### Importance of peers as a source of information about mitigation measures

Many participants discussed the solidarity among fellow residents and how in addition to making their own masks, they often were bearers of change among their peers.

*“The people that I’ve talked to, I would remind them, ‘Mask up, not blow your nose, mask above your nose…. Wash your hands, try not to be me in anybody’s spaces.’ I did try my best to help my friends with stuff around me, and my personal health hygiene as far as that goes.”* – Participant A, a 30-year-old male.

*“Yeah. Yeah, we did. We looked out more so for each other. … And like you know if we hear someone is having a cough or things like that, we were like, “Okay, stay away from her because she doesn’t” you know or if we see somebody who did display any kind of symptoms, we would go behind them like at the water fountain and wipe it down before the next person you know?”* – Participant L, a 45-year-old female.

Others felt they needed to set boundaries with their peers and keep distance from those who were not following guidelines:

*“Every time I see somebody without a mask on I’m like throw my own shirt on my face like, I pretended I was choked by them so people can make sure that they put their masks on. … Instead of making people or having people feeling offended by your words...”* – Participant T, a 39-year-old male.

*“I always try to tell somebody, especially if they were close by me, to stay clean, wear your mask, Because I’m not trying to, but I’m trying to get out. You know what I mean? I am not trying to die in jail.”* – Participant F, a 35-year-old male.

### Discordant perceptions on the safety of correctional settings compared with the community during the pandemic

The limited information received about the pandemic, diminished access to resources and the actions of those whose job it is to protect them, led some participants to report conflicting feelings stemming from their anticipated release.

*“I was afraid to come home to tell you the truth. I wanted to stay there until it was over, because by that time there was only five of us left in the prison, because a lot of guys were being sent home due to underlying medical conditions and they didn’t want to be responsible for anything tragic happening. But while I was there, I’d rather stay there until the pandemic was over. Because then there was riots going on and everything was going crazy.”* – Participant D, a 50-year-old male.

Similar feelings about the surrounding community were expressed by this participant:

*“There’s a lot of people that haven’t been vaccinated, there’s still people that don’t take it serious, they don’t wear their masks in the stores. I know they’ve recently lifted the ban, like there was like a mandatory mask ban, and it seems like when they lifted that everyone just went buck wild, and no one adheres to the six-foot rule. I’m vaccinated, wear a mask, and I’m still constantly saying, “Can you please step back?” Because I mean, you don’t know, but people just feel like, “Oh, they lifted the mask rule, so we’re safe to just go buck wild.” I don’t understand it.”* – Participant B, a 36-year-old female.

While most perceived COVID-19 to be an ongoing threat to themselves as well as community dwelling individuals, limited interactions with individuals with severe COVID-19 led others to be skeptical about the severity of the disease. However, even those with their doubts continued to follow precautions.

*“I’ve had nobody that I know of that has died from it. I have nobody that I know who has been hospitalized about it. … I mean, I don’t see anything really outright, totally kicking everybody’s ass. And I mean maybe the mask thing is working, but I’ve been going around Bridgeport right now and a lot of people aren’t wearing masks, they’re not making you wear masks when you walk in the store, even though there’s a sign outside. And I usually put one on. I go to a methadone program too, so I put one on. I mean I try and act like it, like I care. …”* – Participant Q, a 56-year-old male.

### Wastewater-based surveillance as a logical strategy

The majority of the recently released subjects (68%) believed using WBS to monitor for COVID-19 would work in their last facility and 79% endorsed routinely monitoring SARS-CoV-2 levels through WBS rather than relying solely on individual testing (Table 3). Some indicated that this was the most logical strategy since integrating WBS into current surveillance methods would help prevent larger outbreaks rather than waiting for individuals to exhibit symptoms or test positive.

*“Because I feel that [WBS] would catch it before everything could start. If you do the individual randomly, by the time you do the individual character be spreading, the other way you can catch it before, it’s a spread.”* – Participant F, a 35-year-old male.

*“I think wastewater testing – because it could help with early, early detection.”* – Participant H, a 52-year-old male.

Another participant supported using WBS as a tool for deciding if and when to re-implement COVID-19 restrictions such as quarantining, mask-wearing and social distancing at the facility and community-level.

*“Yeah, I’m all for that [continued WBS]. If you can isolate it right, then and save many of the lives then isolate it. I’m all for it.”* – Participant J, a 59-year-old male.

Others, such as the following participant, expressed preference for using both:

*“They should do it both. … To make it safer. To be actually sure. Because they could do the rapid test or like the rapid test the captain came in and explained to us that it’s a bullshit test. It’s not an official test. Then they will do the long test for two weeks. That’s the actual test. But people still slip through the cracks, you know? They still do it. Look this place is popping back up again but people are still fucking ignorant.”–* Participant O, a 49-year-old male.

When it comes to settings where individual have the option to get tested or not, some participants preferred WBS instead of relying on their peers to take the initiative to get tested.

*“I mean, because everyone goes to the bathroom, and not everyone is going to want to cooperate …So, without anybody really having – you don’t have to get anybody’s permission to check the water, it makes it just a lot easier. … I mean, individual testing is going to be a problem because not everyone is going to want to participate. People are going to give you a hard time and some people are not going to be available or some people are going to make sure that they’re not available to even participate.”* – Participant D, a 50-year-old male.

Despite their support, some participants had their concerns, particularly regarding acceptability and willingness among staff to implement WBS.

*“It just all depends on each facility treats people different, each facility’s correctional like officers or whatever … Some of them will go for that [waste-based surveillance]. Some of them won’t, some of them will want to do it their own way. And not only the facility, you’re talking to each person that works there has their own view on how they want to treat people. …”* – Participant E, a 30-year-old male.

#### Preference for individual testing

While most supported WBS in combination with individual testing, some participants thought individual testing would provide additional benefits. Such as this participant who was concerned some of his peers would perceive the combined strategy as undermining the health of all residents.

*“Like, if I had to choose? I would do individual. I think, because everyone, like matters. So, I think that individual testing will give each person that sense that they actually care for their health is taken into account and they’re not just being allowed to be like, second-hand subjects, just because you’re not testing everybody. So, some people may feel that okay, they’re not being cared for properly, because you’re not testing everybody and so, they believe, make it seem like their lives don’t matter.”* – Participant C, a 31-year-old male.

Others believed individual testing to be more accurate that WBS.

*“I feel like, I don’t know if I would really feel more secure that it’s, that it would be okay just for the wastewater testing as opposed to the individual testing. I just think it would – my personal opinion, I just think it would be more accurate.”* – Participant L, a 45-year-old female.

#### Poor water quality in correctional settings fostering concern about legitimacy of WBS

Despite the overall support to use WBS to monitor for COVID-19 both in their former facility and the surrounding community, there was confusion surrounding this method of testing for some participants. Many emphasized the historically persistent poor quality of the drinking water.

*“The water in prison, they’re not like the water in the streets. Even before the Corona, the whole water you put it in your cup and it’s almost like a cloud color... And when you drink it kind of fizzles down your throat. I’m like what the fuck I’m I drinking? … They already know the water supply was shitty for the past 34 years, I’ve been going in and out of prison you know? So I don’t know.”* – Participant O, a 49-year-old male.

## Discussion

In this study we aimed to understand how to improve infection control in correctional settings and as assess knowledge and attitudes towards using WBS for SARS-CoV-2 surveillance among individuals with lived experience of incarceration during COVID-19 pandemic. This study contributes important data from the perspectives of individuals incarcerated during COVID-19 on infection control measures in carceral settings, perceived ways in which infection control measures could be improved, and perspectives regarding the acceptability of WBS accompanying individual testing. Our findings suggest that while justice-involved individuals do support routine WBS for SARS-CoV-2, participants had concerns about feasibility and acceptability at the facility level. Understanding the acceptability of integrating this method of routine surveillance for SARS-CoV-2 and the related concerns such as correctional facilities being unable to fully implement COVID-19 mitigation measures, correctional staff’s limited adherence to facility guidelines, and the lack of transparency regarding COVID-19 is important to implementing strategies for successful surveillance.

A key finding was how the inconsistent implementation of COVID-19 guidelines and actions of staff perpetuated feelings of insecurity and conveyed a lack of empathy for the residents. In some instances this stemmed from the limited access to COVID-19 resources to such as masks, cleaning supplies and routine testing, were noted during the early stages of the pandemic, particularly among healthcare workers (Livingston et al. 2020; Emanuel et al. 2020)and marginalized populations such as people who use drugs (Schneider et al. 2021; Nelson and Kaminsky 2020; CARCERÁRIA 2020; Carcerári 2020). For example, a Brazilian organization reported that Brazilian correctional facilities were not providing adequate PPE (CARCERÁRIA 2020), and that 66% of food and hygiene materials that individuals sent to their family members who were incarcerated never reached them (Carcerári 2020). Feelings of insecurity also arose regarding the inability to socially distance due to the congregate nature of these facilities, which was reported in the literature as well (Pyrooz 2020). Some of our participants who reported feelings of frustration from resource and space constraints, leveraged these feelings to take the lead in implementing change in their facility. Oftentimes, they took matters into their own hands to manufacture their own protective equipment, while for others these changes were rooted in solidarity and looking out for one another. In other settings were resources were limited, some participants sought out sessions with correctional healthcare staff to learn more about COVID-19 and how to reduce their risk (Pettus-Davis 2021).

While there is substantial evidence that justice-involved individuals often perceive a lack of compassion from the facility-level and staff(Testoni et al. 2021; Testoni et al. 2020; Hobbs and Dear 2000; Trammell and Rundle 2015; Vieraitis 2018), many participants revealed that this feeling was exacerbated by the pandemic. This echoes calls from advocates early in the pandemic that correctional staff should not use COVID-19 restrictions as an excuse to undermine the rights of those whose lives they are responsible for (Crowley et al. 2020). These feelings were also associated with a delay in receiving information among participants, which has been noted in other settings (Pettus-Davis 2021; Carvalho et al. 2020). Early in the pandemic, Carvalho and colleagues called for correctional facilities to be transparent in their dissemination of COVID-19-related information to justice-involved individuals and their families (Carvalho et al. 2020). The need for transparency was echoed by findings from a cohort of over 300 justice-involved individuals in the U.S., in which 69% reported they learned about COVID-19 from the television rather than from their facility (Pettus-Davis 2021). Furthermore, participants in a cohort of individuals incarcerated in the Oregon Department of Corrections during the pandemic suggested that the institutional newsletters that were distributed throughout their facilities were inadequate (Pyrooz 2020).

While less common, we found some skepticism among participants regarding the severity of COVID-19 and trust in the vaccine. This reflects findings from other studies regarding disease outbreaks in carceral settings (Stern et al. 2021; Geana et al. 2021; Chin et al. 2021). For example, just over one-fifth of individuals sampled across 13 U.S. jails were either hesitant or refused to get the vaccine because they did not perceive themselves to be at risk for COVID-19 or perceived vaccination as unnecessary (Stern et al. 2021). Similarly, a common theme that emerged among a qualitative cohort of formerly incarcerated women from the Midwest was the perceived exaggeration of the number of COVID-19 deaths and concern that the COVID-19 vaccines have electronic chips in them (Geana et al. 2021).

Integrating WBS into surveillance measures at their most recent facility was acceptable among most participants. With COVID-19 still perceived an ongoing threat for many, the lack of safety while incarcerated and upon to their return the community generated support for the integration of WBS into current SARS-CoV-2 monitoring methods. However, some indicated preference for individualized testing, favoring opt-in testing for SARS-CoV-2 as it would create a sense of autonomy particularly among a population that often feels as though their health is not held in as high regard as others. This concern has been noted in the context of HIV testing among justice-involved individuals. The implementation of opt-out HIV testing in correctional facilities raised concerns that by nature incarcerated individuals do not have full autonomy and therefore they may not truly understand what type of testing they are receiving (Celada et al. 2011; Seal et al. 2010; Walker et al. 2005; Rosen et al. 2015).

Regardless of preference for opt-in or opt-out testing for SARS-CoV-2, the overall acceptability of integrating WBS into existing surveillance strategies is promising. Increasingly more facilities have the capacity to conduct screening of asymptomatic residents and employees (Hagan et al. 2020); and offer vaccines to these two groups (Kronfli and Akiyama 2021). Mass testing events in carceral settings indicates that the majority of cases identified are either pre-symptomatic or asymptomatic demonstrating the importance of widespread testing in early case identification rather than relying on only testing symptomatic individuals (So 2020; Lemasters et al. 2020). The integration of low-effort, routine WBS into existing protocols for between outbreaks will respect individual autonomy in settings where opt-in testing is preferred while supporting ongoing mitigation efforts, and will complement routine opt-out testing in settings where that is the norm. Therefore, incorporating WBS into existing testing and mitigation strategies is not only the logical next step but the acceptable one as well.

### Limitations

This study is not without its limitations. Participants were referred to the study by partnering organizations that work directly with formerly incarcerated individuals and by research staff who were onsite at corrections-focused CBOs in Georgia, New York, Connecticut, and Massachusetts. To aid in anonymity of participants, we did not ask participants the name of the correctional facility they were housed in during the interview. Moreover, variations in facility sizes across the country and the qualitative nature of our work, our findings may not be generalizable to formerly incarcerated individuals in other regions, individuals in those states who do not receive services from corrections-focused CBOs, or from individuals from those organizations without access to a phone or computer to conduct the interview virtually. Furthermore, individuals were considered eligible if they were incarcerated between March 2020 and the May 2021 some participants were already in a facility prior to the start of the pandemic. Therefore, findings related to testing and vaccine availability may not be generalizable to all individuals incarcerated during this period, since some were not tested upon entry while others were released prior to vaccine availability and were unable to receive the vaccine at their most recent facility. An additional limitation is within the qualitative data. While participants watched a video on WBS prior to being asked about the feasibility and acceptability of using WBS to monitor for SARS-CoV-2, it was apparent that many did not fully grasp the concept, limiting out ability to determine if using WBS testing for surveillance methods is truly acceptable to some participants.

## Conclusion

In conclusion, the COVID-19 pandemic perpetuated existing feelings of insecurity and a perceived lack of empathy from the correctional facility residents, which stemmed from COVID-19 guidelines implemented at the facility level and actions of the staff. Key recommendations to build on this integration include mandating testing upon entry, transparency with residents on the virus and its spread, and understanding facility resources. We also found that routine WBS to monitor for SARS-CoV-2 is acceptable to formerly incarcerated individuals even if it means reimplementing COVID-19 restrictions when the virus is detected in the wastewater supply. Integrating WBS into existing surveillance strategies to monitor for SARS-CoV-2 can help mitigate future outbreaks while simultaneously conserving already constrained resources. As jurisdictions begin to integrate WBS into existing surveillance strategies, it will be important to identify best practices as well as barriers to implementation.

## Data Availability

Not applicable.
